# Prevalence of Depression and Anxiety Symptoms Among Parents of Hospitalized Children in 14 Countries

**DOI:** 10.3390/children12081001

**Published:** 2025-07-30

**Authors:** Linda S. Franck, Renée Mehra, Christine R. Hodgson, Caryl Gay, Jennifer Rienks, Amy Jo Lisanti, Michelle Pavlik, Sufiya Manju, Nitya Turaga, Michael Clay, Thomas J. Hoffmann

**Affiliations:** 1 Department of Family Health Care Nursing, University of California San Francisco, San Francisco, CA 94143, USA; renee.mehra@ucsf.edu (R.M.); caryl.gay@ucsf.edu (C.G.); michelle.pavlik@ucsf.edu (M.P.); sufiya.manju@ucsf.edu (S.M.); nitya.turaga@ucsf.edu (N.T.); michael.clay@ucsf.edu (M.C.); 2 Family and Community Medicine, University of California San Francisco, San Francisco, CA 94143, USA; jenrienks@aol.com; 3 Department of Family and Community Health, School of Nursing, University of Pennsylvania, Philadelphia, PA 19104, USA; lisanti@nursing.upenn.edu; 4 Research Institute, Children’s Hospital of Philadelphia, Philadelphia, PA 19146, USA; 5 Department of Epidemiology and Biostatistics, University of California San Francisco, San Francisco, CA 94143, USA; thomas.hoffmann@ucsf.edu

**Keywords:** parent, primary caregiver, hospitalized child, mental health, anxiety, depression, social drivers of health, housing insecurity, discrimination in daily life, child health, family-centered care, social support, self-care

## Abstract

**Highlights:**

**What are the main findings?**

- In this cohort of 3350 parents from 14 countries, about half reported depression symptoms and over two-thirds reported anxiety symptoms.
- Poorer rating of their child’s health was associated with an increased risk of depression and anxiety symptoms, whereas higher levels of self-care and social support were associated with a reduced risk of depression and anxiety symptoms.

**What are the implications of the main findings?**

- Depression and anxiety symptoms in parents of hospitalized children are common globally.
- Hospitals should consider routine symptom screening and preventative mental health and support services for parents as part of standard care for hospitalized children.

**Abstract:**

**Background/Objectives**: Despite the importance of parent mental health for child health, there are no global prevalence data on parental mental health symptoms when children are hospitalized. We aimed to describe depression and anxiety symptom prevalence and associated factors among parents of hospitalized children. **Methods**: We conducted this 14-country prospective cohort survey with parents/primary caregivers staying at a nearby Ronald McDonald House^®^ during their child’s hospital treatment. We used the Hospital Anxiety and Depression Scale to measure depression and anxiety symptoms and validated scales and theory-based questions to measure parent, family, and child covariates. We calculated the prevalence of clinically significant or concerning symptoms of depression and anxiety, and used multivariable regression analyses to examine associations between covariates and outcomes. **Results**: Among 3350 participants, 1789 (49.7%) reported depression symptoms and 2286 (69.0%) reported anxiety symptoms. Worry about housing and poorer ratings of their child’s health were associated with increased risk of depression symptoms. Poorer rating of the child’s health, living with a partner, and discrimination in daily life were associated with increased risk of anxiety symptoms. Higher levels of self-care, hospital family-centered care, and social support were associated with reduced risk of depression symptoms. Higher levels of self-care and social support were associated with reduced risk of anxiety symptoms. **Conclusions**: Clinically significant or concerning depression and anxiety symptoms are common among parents of hospitalized children globally. Hospitals should consider offering routine mental health symptom screening and preventative mental health and support services to all parents.

## 1. Introduction

Globally, millions of children receive hospital treatment each year, which can be a traumatic experience for the entire family. In the short term, families experience stress, distress, worry, loss, grief, and may face difficulties in meeting their basic needs such as sleep, nutrition, space to move, and financial costs [1,2,3,4,5]. Children who are in the hospital experience fear, worry, sadness, boredom, and loneliness [6]. A child’s hospitalization for serious illness can lead to long-term physical and mental health problems for the entire family, including anxiety, depression, and financial hardship that adversely affect children’s health and development after they return home [1,2,3,4]. Parents of hospitalized children experience significant psychological distress, including depression, anxiety, and post-traumatic stress symptoms [7]. The stress is compounded for those who must travel long distances to the hospital to provide essential care and support to their hospitalized children while maintaining the welfare of other children and family members at home [8,9]. There are limited pediatric hospital interventions focused on parents’ mental health, but evidence has shown that family-centered models of care, psychosocial interventions, and practical support to assist parents and families in meeting basic needs can buffer the adverse effects of a child’s hospitalization [10,11,12,13]. Family-centered care is an approach to healthcare that is grounded in mutually beneficial partnerships among patients, families, and healthcare teams, and results in higher quality and safer healthcare [14]. There is some evidence that coping support interventions, which include education, emotional regulation, social support, or a combination thereof, are associated with lower parent stress and anxiety [10].

Despite the importance and scale of the threat to parental well-being, research is limited, often focusing on specific diagnostic groups, conducted in single centers, or using retrospective designs. Moreover, since the 2020 COVID-19 pandemic, the mental health of parents worldwide has worsened [15]. However, there are no contemporary global data on the mental health of parents of hospitalized children. To address this knowledge gap, we partnered with Ronald McDonald House Charities^®^ (RMHC^®^) to conduct research to better understand the mental health, socioeconomic needs, strengths, and healthcare experiences of families of hospitalized children. RMHC is a non-profit organization with Chapters in 62 countries and regions that provide temporary accommodation, meals, peer support, and other services to meet the basic needs of families of children receiving hospital care (https://rmhc.org; accessed on 26 July 2025) [16]. The aims of this study were to describe the depression and anxiety symptoms experienced by parents of hospitalized children and to examine relationships between depression and anxiety symptoms and parent, family, and child demographic and socioeconomic characteristics, and healthcare experiences.

## 2. Materials and Methods

### 2.1. Study Design and Participants

The Learning From Families study is a global study with parents who stayed at a Ronald McDonald House^®^ during their child’s hospital treatment. A research advisory group consisting of RMHC leaders, subject matter experts, and parent advisors provided input throughout the study planning and implementation. Participants were recruited from 34 Ronald McDonald Houses in 14 countries. Study sites were selected from countries served by RMHC based on the site’s interest and capacity to engage in research and varied with respect to size and population served. Ethical review and approval were waived for this study by the University of California, San Francisco Committee on Human Research (20-31401). Recruitment occurred between March 2023 and March 2024, with data collection for the survey completed in April 2024. This study followed the Strengthening the Reporting of Observational Studies in Epidemiology (STROBE) reporting guideline for reporting observational studies [17].

Parents (defined as the biological parent or other primary caregiver) 18 years of age or older of children receiving hospital treatment and who stayed at a participating Ronald McDonald House for at least three consecutive days, and who spoke one of the available survey languages, were eligible to participate. Only one parent for each child was eligible, and those with repeated stays participated only once. Eligible individuals interested in the study signed a Ronald McDonald House permission form to have their contact information shared with the study team. These potential participants received an invitation with standard consent information and the enrollment survey in one of up to three languages at each site. Participants indicated agreement to participate by completing the survey after reviewing the study information sheet. The survey was provided in eight languages: English, French, Italian, Japanese, Portuguese, Spanish, Tagalog, and Traditional Chinese (up to three languages at each site) via email, text, or paper, depending on participant preference. Participants received the equivalent of a $20 USD gift card or gift for each completed survey.

### 2.2. Primary Outcomes

Parental depression and anxiety symptoms experienced over the two weeks prior to survey completion were reported using the internationally validated Hospital Anxiety and Depression Scale (HADS) [18]. The HADS is one of the most widely used and well-validated measures of depression and anxiety and has been translated into over 100 languages [19]. It has 14 items and two subscales measuring symptoms of anxiety (7 items) and depression (7 items). Each item is rated on a 4-point, 0–3 scale [20,21]. Higher scores indicate more severe/frequent symptoms. Total subscale scores 0–7 on each subscale are considered “normal”. Scores 8–10 suggest borderline anxiety (HADS anxiety subscale) or depressive (HADS depression subscale) symptoms, and scores > 10 are considered “abnormal (cases).” Scores in the “borderline” and “abnormal” range (scores ≥ 8) represent clinically significant or concerning symptoms of anxiety or depression [21]. The HADS is often used in research to measure symptoms of anxiety and depression in parents of hospitalized infants and children [5,16,22].

### 2.3. Covariates

The selection of covariates was guided by the National Institute of Minority Health and Health Disparities Research Framework (NIMHD) [23] to ensure the survey was inclusive of social drivers of health relevant to understanding health and health disparities while minimizing the research burden on participants (Table S1). The survey content was pilot-tested with input from local study advisors to ensure linguistic and cultural appropriateness and relevance. Parent characteristics and social drivers of health included age, relationship to the hospitalized child, education level, health literacy, marital/relationship status, serving as a primary caregiver for others besides children, number of own health concerns or disabilities, employment status, type of health insurance, prior experience of discrimination in daily life, frequency of basic self-care in the prior month (i.e., sleep, nutrition, exercise, etc.), and social support. Family characteristics included household size, geographic region, income, number of unmet basic needs, and worry about not having stable or safe housing. Child characteristics included age, number of health concerns or disabilities, and parental rating of the child’s current health. Healthcare experiences included the length of the Ronald McDonald House stay at the time of survey completion, whether it was a first-time stay at a Ronald McDonald House, travel time between the family’s home and hospital, approximate hours/day spent with the child at the hospital, having more than one child receiving hospital care, perception of the family-centeredness of hospital care, and perception of the family-centeredness of services at the Ronald McDonald House.

### 2.4. Statistical Analysis

Descriptive statistics were calculated using survey sample weights. Per recommendations for handling missing scale data, if an individual was missing any scale questions (e.g., a question from the HADS depression scale), the participant-specific mean was multiplied by the number of questions and used [24]. A univariable survey sample-weighted relative risk log–linear model was implemented through a Poisson regression model with robust standard errors for each of the covariates of interest and the outcomes. To determine independent associations with the outcomes, variables with *p* < 0.2 were included for consideration in a backwards stepwise multivariable model. This approach allowed for the systematic elimination of variables that did not significantly contribute to the model, leading to a more parsimonious and interpretable final set of independent associations from the initially comprehensive candidate list. See Supplementary Materials Methods S1 for additional methodological details, including sample size estimation and computational details of the backwards stepwise regression approach with multiple imputations.

## 3. Results

### 3.1. Parent, Family, and Child Characteristics

**Figure 1:**
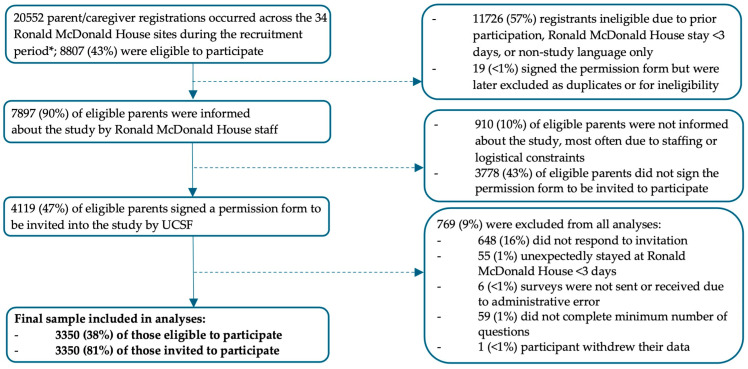
Participant flowchart.* Study recruitment periods varied by Ronald McDonald House site and ranged from 3 to 12 months. Two of the thirty-four participating sites (6%) dropped out of the study prior to its completion.

Of the 20,552 parents registered at participating Ronald McDonald Houses during the study period, 8807 (43%) were eligible to participate in the study (Figure 1). Of these, 7897 (90%) were informed about the study, 4119 (47%) signed a permission form to be invited to the study, and 3350 (38%) completed the survey and were included in the analysis. The survey completion rate was notably high (e.g., an average of 3.6% missingness for the characteristics included in the regression analyses).

Parent, family, and child characteristics are presented as unweighted counts and weighted percents, means, or medians unless otherwise noted (Table 1; additional characteristics in Table S2). There were 2594 (78.6%) mothers, 616 (18.1%) fathers, and 131 (3.3%) other caregivers included in the study. The number of parents who stayed at a Ronald McDonald House in the United States (US) was 1538 (69.0%), 995 (11.7%) in Latin America, 486 (9.7%) in Canada, 183 (6.2%) in the Asia-Pacific region, and 148 (3.4%) in Europe. The median age of the hospitalized child was 3 years (interquartile range [IQR] 0.1, 11). The three most common reasons for the child’s hospital treatment were the following: cardiology (846, 24.3%), neonatal (679, 23.1%), and oncology/hematology (750, 20.9%).

The majority of parents lived 2 or more hours from the hospital (2251; 70.5%). However, while staying at the Ronald McDonald House, 2452 (82.2%) were able to spend 7 or more hours per day on average with their hospitalized child. About half of the parents (1789; 49.7%) had clinically significant or concerning symptoms of depression and 2286 (69.0%) had clinically significant or concerning symptoms of anxiety (Table 2), with 1514 (43.8%) experiencing clinically significant or concerning symptoms of both depression and anxiety.

### 3.2. Factors Associated with Depression Symptoms

**Figure 2:**
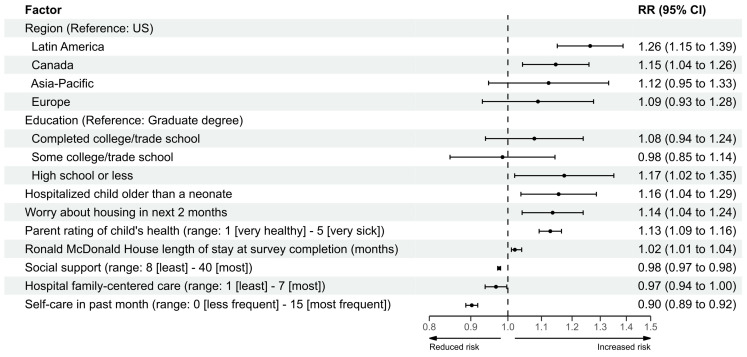
Forest plot of multivariable regression model analysis for depression symptoms. RR: relative risk; CI: confidence interval; US: United States.

In multivariable regression analyses, having a hospitalized child older than a neonate (relative risk [RR]: 1.16; 95% confidence interval [CI]: 1.04–1.29), being worried about housing in the next two months (RR: 1.14; 95% CI: 1.04–1.24), perceiving their child as having poorer health (RR: 1.13; 95% CI: 1.09–1.16), and having a longer stay at the Ronald McDonald House before completing the survey (RR: 1.02; 95% CI: 1.01–1.04) were each associated with increased risk of depression symptoms (Figure 2). Compared to having a graduate degree, having high school or less education was associated with an increased risk of depression symptoms (RR: 1.17; 95% CI: 1.02–1.35). Parents of children hospitalized in Latin America (RR: 1.26; 95% CI: 1.15–1.39) or Canada (RR: 1.15; 95% CI: 1.04–1.26) compared to those in the US had a higher risk of depression symptoms. More frequent self-care in the past month (RR: 0.90; 95% CI: 0.89–0.92), higher rating of hospital family-centered care (RR: 0.97; 95% CI: 0.94–0.997), and social support (RR: 0.98; 95% CI: 0.97–0.98) were each associated with reduced risk of depression symptoms (Figure 2).

### 3.3. Factors Associated with Anxiety Symptoms

**Figure 3:**
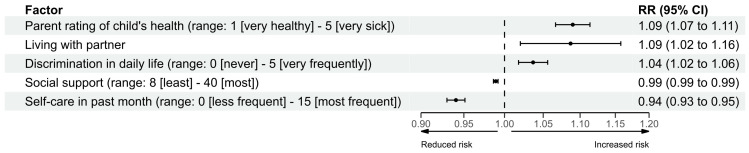
Forest plot of multivariable regression model analysis for anxiety symptoms. RR: relative risk; CI: confidence interval.

Perceiving their child as having poorer health (RR: 1.09; 95% CI: 1.07–1.11), living with a partner compared to not living with a partner (RR: 1.09; 95% CI: 1.02–1.16), and more frequent experiences of discrimination in daily life (RR: 1.04; 95% CI: 1.02–1.06) were each associated with increased risk of anxiety symptoms (Figure 3). More frequent self-care in the past month (RR: 0.94; 95% CI: 0.93–0.95) and higher levels of social support (RR: 0.99; 95% CI: 0.986–0.992) were each associated with reduced risk of anxiety symptoms.

## 4. Discussion

This is the first prospective global survey of depression and anxiety symptoms and associated factors among parents of children receiving hospital treatment. In contrast to prior studies with parents of children with special healthcare needs or life-limiting illnesses [1,2,3,4], this study included any primary caregiver of a child admitted to any pediatric unit for any health condition, providing new insights into the overall high prevalence of parental depression and anxiety symptoms, and factors associated with depression and anxiety symptoms. In prior meta-analyses among parents of children with chronic conditions, the weighted mean prevalence rate of depression and anxiety was 20.9% and 16.0%, respectively, or about three times that of the general population for depression and about twice that for anxiety [25,26]. In this study, although over half (57.2%) of the children had a chronic health concern or disability, the prevalence of depression and anxiety symptoms was much higher, 49.7% and 69.0%, respectively. These are similar to the symptom prevalence among parents of critically ill children or those with cancer and indicate significant caregiver burden and potential risk for adverse consequences for the child and family if unaddressed [1,2,3,9,10]. Some regional differences in the prevalence of parental depression symptoms were noted, although not for anxiety symptoms. The prevalence of mental health conditions worldwide is known to vary by country and has been on the rise for some years, exacerbated by the COVID-19 pandemic [15,27]. Consistent with the multiple domains and levels of influence affecting health outcomes in the NIMHD Research Framework [23], we found associations between parental depression and anxiety and the covariates measured across the behavioral, sociocultural environment and healthcare system, and across individual and interpersonal levels. Our greater understanding of these relationships can inform efforts to strengthen health and social systems, as discussed below.

### 4.1. Social Drivers of Health

The increased prevalence of depression symptoms globally is partially attributed to worsening socioeconomic factors and social isolation [28]. Several of these social drivers of health were associated with increased risk of depression and anxiety symptoms in this study. Housing instability has been associated with poorer parental physical and mental health, including maternal depression, and poor child health, including more frequent rehospitalizations [29]. In this study, most parents lived far from the hospital, almost half reported incomes at or below the poverty level for their country, and 35% reported a range of unmet basic family needs. Having a child with a life-threatening or chronic illness puts further financial strain on the family [30]. Worry about housing in the next two months was the most salient economic factor associated with depression symptoms. Lower parent education was associated with an increased risk of depression symptoms, consistent with prior findings among mothers of hospitalized preterm infants [31]. In contrast, no relationship was found between education level and anxiety symptoms in this study. Prior studies have reported inconsistent findings regarding the associations between education and anxiety among parents of neonates [31,32] and critically ill children [33]. Prior discrimination in daily life was associated with a greater risk of anxiety symptoms. This is consistent with a systematic review of international studies [34] and a recent US study that found associations between experience of discrimination and poorer adult mental health, including depression and anxiety [35].

### 4.2. Child Illness

Worse parental rating of child health was associated with an increased risk of both depression and anxiety symptoms and longer stays at a Ronald McDonald House was associated with increased risk of depression symptoms. While these factors may not be directly modifiable, early intervention to support parents who perceive their children to be very ill and those with extended hospital stays may prevent long-term mental health problems and negative impacts on children and families [10,11].

### 4.3. Social Support, Basic Self-Care, and Hospital Family-Centered Care

Parents who reported higher levels of social support had lower risk of depression and anxiety symptoms. Social support, including peer support, has been consistently found to buffer the effects of a child’s illness on parental mental health [36,37]. While having a partner is often considered a form of social support, in this large cohort it was associated with an increased risk of anxiety symptoms. A child’s hospital treatment and chronic illness can be sources of stress in partner relationships, especially when there is pre-existing relationship strain or decisional conflict [37]. However, social support from family and friends is associated with better mental and physical health for mothers of critically ill children, even in the context of low spousal support [38].

Parents who reported more frequent basic self-care in the past month, including sleep, hydration, nutrition, and exercise, had a lower risk of depression and anxiety symptoms. Self-care is known to be important for the sustainment of family caregiver mental health across all ages and conditions [39]. Parents who reported higher levels of hospital family-centered care had a lower risk of depression symptoms. This finding aligns with a large body of research supporting family-centered care as a standard of care in pediatric hospitals, which is associated with better parental mental health and the health and development of the hospitalized child [11,12,13].

### 4.4. Strategies to Address the High Prevalence of Parental Depression and Anxiety

Several strategies can be taken to immediately address the factors associated with parental depression and anxiety identified in this study. Health and social service professionals must be made aware of the high prevalence of parental depression and anxiety symptoms. Awareness must be translated to action by engaging parents in designing methods to sensitively inquire about social drivers of health that may be affecting families, such as housing instability and prior discrimination, and referral to appropriate social services. Cross-sector collaboration is essential to address the social needs of families. Implementing evidence-based policy initiatives can improve the health of families and reduce disparities [40]. This work includes expanding access to support services, such as social support and basic self-care, which are highly valued by parents [41]. Without support services, parents are at risk for long-term psychosocial, physical, and financial hardship that can affect the health and well-being of all family members. Community-based organizations are particularly well-positioned to address parental social support and self-care needs, helping to mitigate urgent material hardship related to the child’s hospitalization by providing overnight accommodation, meals, peer support, and other services to meet basic needs [11]. While global and inter-agency initiatives have begun to invest in supportive interventions for parents [42], coordinated efforts are needed to address the mental health and social needs of parents of hospitalized children.

### 4.5. Global Research Gaps

The study findings are also a call to action to the global research and child health advocacy communities to coordinate efforts to address silos in child and family health metrics and improve global data availability. Given the scale of the problem of parental depression and anxiety, it is astonishing that there are no publicly accessible data on how many of the world’s 2.3 billion children are hospitalized each year. Nor are there standard metrics for parent and family outcomes following child hospitalization. Data are scarce and predominantly from high-income countries. Greater global collaboration is needed to address the research gaps and gain a comprehensive worldwide understanding of the prevalence, risk, and protective factors for mental health among parents of children receiving hospital care [43]. Research is also needed to better understand the trajectory of parental depression and anxiety symptoms in the months and years following the child’s discharge from the hospital to inform recommendations for screening and referral for treatment. Beyond a better understanding of the problem, comparative intervention studies are urgently needed to ascertain the most effective and scalable interventions to reduce parental depression and anxiety and improve child, parent, and family health during and following a child’s hospitalization.

### 4.6. Limitations and Strengths

The study findings should be considered in light of several limitations and strengths. The sample was recruited from Ronald McDonald Houses, and the findings may not be generalizable to parents who stayed exclusively at their child’s bedside or elsewhere. However, about one-third of the survey respondents were wait-listed prior to staying at a Ronald McDonald House, and their perspectives are included in these findings, providing representation of parents who stayed elsewhere during their child’s hospitalization. In addition, parents who stay at a Ronald McDonald House more often live farther away from the hospital and have children with serious medical conditions, often requiring longer lengths of stay, which may also limit the generalizability of the findings. All data collected for this study were from parent report and no hospital clinical data, including clinician-assessed severity of a child’s illness, were collected. However, clinician-assessed severity of a child’s illness has been shown not to be associated with parental mental health, whereas parental perception of the child’s illness is associated with parental mental health symptoms [36]. Although this is the largest and only multi-country study to date, many countries are not represented, and we found some regional differences in depression symptoms that were not explained by the covariates examined. Further multi-country and regional research is needed. The prevalence and severity of depression and anxiety symptoms among study participants prior to their child’s hospitalization are unknown, and therefore, the prevalence of symptoms specifically attributable to the traumatic stress related to the child’s hospital admission cannot be determined from this study. However, regardless of whether the mental health symptoms were pre-existing or new, a significant proportion of parents suffered clinically significant or concerning levels of symptoms during their child’s hospitalization, and this warrants greater attention to preventative and supportive interventions. Finally, although the survey examined a wide range of potential factors influencing parental depression and anxiety, there are likely unmeasured factors to consider in future research. Strengths of this study include the parent and community partnership in the design and implementation; the diversity of the countries, languages, characteristics of participants, families, and children; and the completeness of the data. These factors increase the generalizability of the findings.

## 5. Conclusions

In summary, the study findings indicate that clinically significant and concerning depression and anxiety symptoms are prevalent among parents of hospitalized children. The breadth of the problem across countries, hospitals, and clinical conditions warrants universal preventative support services, screening, and referral for treatment if needed. The findings are a call to action for healthcare organizations, social service agencies, and community-based charitable organizations to work together to intervene to reduce risks and increase protective factors to address the mental health crisis among parents of children receiving hospital treatment.

## Figures and Tables

**Table 1:** Parent, family, and child characteristics (n = 3350).

Characteristic (Unweighted n)	Unweighted n	Weighted Percent, Unless Otherwise Noted ^a^
**Parent Characteristics and Social Drivers of Health**		
Age (years), median (IQR)	3256	35.0 (29.0, 42.0)
Parental role (n = 3341)		
Mother	2594	78.6%
Father	616	18.1%
Other	131	3.3%
Education (n = 3328)		
High school or less	1436	35.3%
Some college/trade school	560	22.6%
Completed college/trade school	1014	29.9%
Graduate degree	318	12.2%
Living with partner (n = 3324)	2511	76.3%
Discrimination in daily life, median (IQR) ^b^	3312	1.0 (0.0, 2.0)
Frequency of basic self-care in the past month, mean (SD) ^c^	3300	5.9 (2.9)
Social support, median (IQR) ^d^	3279	34.0 (27.0, 39.0)
**Family Characteristics**		
Low household income (n = 2733) ^e^	1380	46.6%
One or more unmet basic needs (n = 3190)	1396	34.8%
Worry about housing in the next 2 months (n = 3280)	834	17.0%
**Child Characteristics**		
Child’s age (years), median (IQR)	3088	3.0 (0.1, 11.0)
Child’s sex or gender (n = 3265)		
Male	1759	54.0%
Female	1470	44.8%
Other	36	1.2%
Having 1 or more chronic health concerns or disabilities (n = 3172)	1743	57.2%
Most common reasons for child’s hospital treatment (n = 3303)		
Cardiology	846	24.3%
Neonatal	679	23.1%
Oncology/Hematology	750	20.9%
Parent rating of child’s current health, median (IQR) ^f^	3027	3.0 (2.0, 4.0)
**Parental Healthcare Experiences**		
Ronald McDonald House length of stay at the time of survey (months), median (IQR)	3292	0.1 (0.07, 0.3)
Travel time between the family’s home and the hospital ≥2 h each way (n = 3299)	2251	70.5%
Time spent with the child in hospital ≥7 h per day (n = 3152)	2452	82.2%
Hospital family-centered care, mean (SD) ^g^	3162	6.2 (1.0)

^a^ Data are unweighted n (weighted %), except age, discrimination in daily life, social support, child’s age, parent rating of child’s current health, and Ronald McDonald House length of stay at the time of survey, which are weighted median (IQR), and frequency of basic self-care in the past month, and hospital family-centered care, which are weighted mean (SD). ^b^ Discrimination in daily life ranges from 0 (never) to 5 (very frequently). ^c^ Frequency of basic self-care in the past month ranges from 0 (less frequent) to 15 (more frequent). ^d^ Social support ranges from 8 (least) to 40 (most). ^e^ Low household income is approximately at or below the poverty line: Argentina—it is hard to pay for basic needs like food and shelter (yes or partially), Australia < $40,000 AUD annually, Brazil ≤ 1 minimum monthly wage, Canada < $50,000 CAD annually, Colombia < 1,000,000 pesos monthly, Ecuador < $450 USD monthly, France < 30,000 Euros annually, Japan < JPY 2,000,000 annually, Italy ≤ 20,000 Euros annually, Peru ≤ S/1000 monthly, Portugal < 12,000 Euros annually, Spain < 12,450 Euros annually, Taiwan < NT$800,000 annually, and United States < $40,000 USD annually. ^f^ Parent rating of child’s current health ranges from 1 (very healthy) to 5 (very sick, fragile, or injured). ^g^ Hospital family-centered care mean ranges from 1 (least) to 7 (most).

**Table 2:** Descriptive statistics for Hospital Anxiety and Depression Scale (HADS) depression and anxiety symptoms.

Outcome (Unweighted n)	Unweighted N	Weighted Percent
**HADS depression (n = 3304)**		
Clinically significant or concerning symptoms	1789	49.7%
No clinically significant or concerning symptoms	1515	50.3%
**HADS anxiety (n = 3304)**		
Clinically significant or concerning symptoms	2286	69.0%
No clinically significant or concerning symptoms	1018	31.0%

HADS depression and anxiety scores were categorized using the standard cutoffs to indicate clinically significant or concerning symptoms (scores ≥ 8) or not (scores < 8).

## Data Availability

Deidentified data will be shared for academic research purposes upon reasonable request directed to Linda S. Franck (linda.franck@ucsf.edu) from qualified investigators up to 3 years after publication. The data are not publicly available due to privacy restrictions.

## Supplementary Materials

The following supporting information can be downloaded at: https://www.mdpi.com/article/10.3390/children12081001/s1, Table S1: Key Covariate Measures: Items, Scales and Sources; Methods S1: Statistical Analysis Methods; Table S2: Additional Parent, Family, and Child Characteristics (n = 3350). References [44,45,46,47,48,49,50,51,52,53,54,55,56,57,58,59] are cited in supplementary materials

## Abbreviations

The following abbreviations are used in this manuscript:

RMHC	Ronald McDonald House Charities
STROBE	Strengthening the Reporting of Observational Studies in Epidemiology
USD	United States Dollar
HADS	Hospital Anxiety and Depression Scale
US	United States
IQR	Interquartile range
SD	Standard deviation
AUD	Australian Dollar
CAD	Canadian Dollar
S/	Sol
JPY	Japanese Yen
NT	New Taiwan Dollar
RR	Relative risk
CI	Confidence interval
