# Supplementary material for: Prevalence of Depression and Anxiety Symptoms Among Parents of Hospitalized Children in 14 Countries
Source: Children (Basel). 2025 Jul 30;12(8):1001. doi: 10.3390/children12081001 (PMC12384160; doi:10.3390/children12081001)
Supplement: Supplementary file 1 [file children-12-01001-s001.zip › children-3748893-supplementary.pdf]

Prevalence of depression and anxiety symptoms among parents of hospitalized children in 14 countries

[insert journal citation when available]

Table of contents

Table S1: Key Covariate Measures: Items, Scales and Sources
Methods S1: Statistical Analysis Methods
Table S2: Additional Parent, Family, and Child Characteristics (n=3350)

Table S1: Key Covariate Measures: Items, Scales and Sources

Covariate	Items/Scale	Source
Discrimination in daily life	6-point scale: "In your daily life, how often have you felt treated or judged unfairly because of your race, ethnicity, religion, sexual orientation, or disability?"; Scoring 5=Very frequently to 0=Never	FAMNEEDS[1]
Frequency of basic self-care in past month	5 items: "How often in this past month have you… Had a good night's sleep; Exercised; Taken time to do something for yourself; Eaten a balanced diet; Drank plenty of water"; Scoring: 0=Never to 3=Always; Total score range: 0 (less frequent) to 15 (more frequent)	Developed for this research with family and community stakeholder co-design
Social support	8 items; Scoring: 1="much less than I would like" to 5="as much as I would like"; Total score range: 8 (least) to 40 (most)	8-Item Duke/UNC Functional Social Support Questionnaire (DUFSSQ)[2,3]
Unmet basic needs	9 items: "In the past year, have you or any family members you live with been unable to get any of the following when it was needed? Food; Clothing, shoes; Utilities; Child's needs; Childcare; Care for an elderly or sick adult; Phone or Internet; Transportation or gas for vehicle; Other" (check all that apply); Total count; 0=No unmet needs	Adapted from: PRAPARE[4]
Worry about housing in next 2 months	"Do you worry that in the next 2 months you/your family may not have a safe or stable place to live?"; Yes or No	FAMNEEDS[1]
Parent rating of child's current health	5-point, Likert-type scale: "How would you (not your child's medical team) rate your child's health now?"; Scoring: 1=Very healthy to 5=Very sick, fragile, or injured.	Adapted from[5]
Hospital family-centered care	7 items; Total score range: 1 (least) to 7 (most)	Adapted from DigiFCC-P[6]

Methods S1: Statistical Analysis Methods

All study data were collected and managed using the REDCap electronic data capture platform (Nashville, TN, USA).[7] All analyses were done in R v4.4.2 (Vienna, AT).[8]

Sample Size Estimation

We initially estimated that 2,000 participants would be needed to detect a clinically meaningful difference in the HADS depression scale for a dichotomous covariate with prevalence 0.1-0.5, assuming 80% power, and a Bonferroni adjustment for nine covariates (α=0.0056). To estimate power, we conducted simulations to determine the minimum detectable effect for a series of sample sizes. Each simulation assumed a standard normal outcome (i.e., we first calculated the standardized effect sizes), a dichotomous covariate, and a site effect (drawn from a standard normal), and then fit a linear mixed effects model with a random site effect. The standardized effect size was then scaled to the HADS depression scale using an SD of 3.075, which was estimated as the average from previous research.[9] A covariate with prevalence 0.1, 0.3, and 0.5, could detect a HADS depression scale (standardized) effect of 0.83 (0.27), 0.55 (0.18), 0.49 (0.16), or larger; i.e., a small-medium effect. A single point on the HADS depression scale is a movement upward/downward on any of the questions summed in the scale. We adopted a more conservative effect size as the power could be reduced because we expected the normality of the depression scale not likely to hold, anticipated potentially categorizing the depression scale score (which we did), and expected incorporating survey weights. The target enrollment for each site was then based on the size of the RMH and the date they started recruitment, which ranged from 50 to 300. To increase the diversity of our sample, we increased our enrollment target to 4,000 families, stopping at 3,406 participants with survey data due to timing.

Missing Data

While the rate of missingness for the fields shown in Table 1 was just 3.6%, additional steps were needed to include the full sample in a multivariate model. To address this, we utilized 500 multiple imputations with multivariate imputation by chained equations (mice) v3.16.0;[10] an additional random seed was run to ensure this number of imputations was reasonable, i.e., that results were stable. Weights were included as covariates in the imputation model.[11]

Survey Sample Weights

Sampling was stratified by sites within countries. We calculated sampling weights by using population size estimates from 2023 records at each site of the number of families with an overnight stay. Specifically, the probability of an individual in the population was calculated as $Pr(s|c) \times Pr(c) = (n_s/\sum_{s'} n_{s'})/(n_c/\sum_{c'} n_{c'})$, where s indexes the sites, c indexes the countries (and includes sites not sampled), and n is the corresponding number of overnight stays. The weight for the site was then given as the probability in the population over the probability of being sampled. Finally, others have noted that setting bounds for trimming weights are not completely agreed upon and there is some arbitrariness to choosing them;[12,13] we trimmed any weight >3.5 times the median weight back to 3.5 times the median weight.[14]

Regression Models

Regression models were fit using the R package survey v4.4.2.[14] To implement a computationally efficient backward stepwise selection model, we followed the approach of Wood et al.[15] Briefly, in this approach, we first fit a stepwise regression model in each imputed dataset. Variables selected in at least half of the fits were retained, and then subject to a backwards stepwise regression (p<0.05) according to the D1 Wald test. We considered 27 covariates for inclusion in regression modeling and we would expect a small number of covariates to appear statistically significant by chance alone. The Bonferroni corrected p-value

threshold would be 0.0019 and many of the significance levels of covariates in the multivariable regression models are below this threshold.[16]

References

1. Uwemedimo OT, May H. Disparities in Utilization of Social Determinants of Health Referrals Among Children in Immigrant Families. Front Pediatr. 2018;6:207. doi:10.3389/fped.2018.00207
2. Epino HM, Rich ML, Kaigamba F, et al. Reliability and construct validity of three health-related self-report scales in HIV-positive adults in rural Rwanda. AIDS Care. 2012;24(12):1576-1583. doi:10.1080/09540121.2012.661840
3. Broadhead WE, Gehlbach SH, de Gruy FV, Kaplan BH. The Duke-UNC Functional Social Support Questionnaire. Measurement of social support in family medicine patients. Med Care. 1988;26(7):709-723. doi:10.1097/00005650-198807000-00006
4. National Association of Community Health Centers, Inc., Association of Asian Pacific Community Health Organizations, and the Oregon Primary Care Association. PRAPARE® Screening Tool. Accessed April 25, 2025. https://prapare.org/the-prapare-screening-tool/
5. Lisanti AJ, Demianczyk AC, Costarino A, et al. Skin-to-Skin Care is associated with reduced stress, anxiety, and salivary cortisol and improved attachment for mothers of infants with critical congenital heart disease. J Obstet Gynecol Neonatal Nurs. 2021;50(1):40-54.
6. Separation and Closeness Experiences in Neonatal Environment (SCENE) research group. Parent and nurse perceptions on the quality of family-centred care in 11 European NICUs [published correction appears in Aust Crit Care. 2017 Jan;30(1):53-54. doi: 10.1016/j.aucc.2016.12.002.]. Aust Crit Care. 2016;29(4):201-209. doi:10.1016/j.aucc.2016.09.003
7. Harris PA, Taylor R, Minor BL, et al. The REDCap consortium: Building an international community of software platform partners. J Biomed Inform. 2019;95:103208. doi:10.1016/j.jbi.2019.103208
8. R Core Team. R: A language and environment for statistical computing. R Foundation for Statistical Computing, Vienna, Austria. Accessed April 25, 2025. Available from: https://www.R-project.org/
9. Franck LS, Shellhaas RA, Lemmon ME, et al. Parent Mental Health and Family Coping over Two Years after the Birth of a Child with Acute Neonatal Seizures. Children (Basel). 2021;9(1):2. doi:10.3390/children9010002
10. van Buuren S, Groothuis-Oudshoorn K. mice: Multivariate imputation by chained equations in R. J. Stat. Soft. [Internet]. 2011 Dec. 12;45(3):1-67. Accessed April 25, 2025. Available from: https://www.jstatsoft.org/index.php/jss/article/view/v045i03
11. De Silva AP, De Livera AM, Lee KJ, Moreno-Betancur M, Simpson JA. Multiple imputation methods for handling missing values in longitudinal studies with sampling weights: Comparison of methods implemented in Stata. Biom J. 2021;63(2):354-371. doi:10.1002/bimj.201900360
12. Valliant R, Dever J, Kreuter F. *Practical tools for designing and weighting survey samples*. 2nd edition. New York: Springer; 2018.
13. National Center For Education Statistics. NAEP weighting procedures: 2003 weighting procedures and variance estimation. Tech. rep. Accessed April 25, 2025. Available from: https://nces.ed.gov/nationsreportcard/tdw/weighting/2002_2003/weighting_2003.aspx
14. Lumley T. Analysis of Complex Survey Samples. J. Stat. Soft. [Internet]. 2004 Apr. 15;9(8):1-19. Accessed April 25, 2025. Available from: https://www.jstatsoft.org/index.php/jss/article/view/v009i08
15. Wood AM, White IR, Royston P. How should variable selection be performed with multiply imputed data?. Stat Med. 2008;27(17):3227-3246. doi:10.1002/sim.3177
16. Miller RG. *Simultaneous Statistical Inference*. Springer; 1966.

Table S2: Additional Parent, Family, and Child Characteristics (n=3350)

Characteristic (unweighted n)	Unweighted N	Weighted percent, unless noted[a]
**Parent Characteristics and Social Drivers of Health**		
Needs help understanding health information (n=3321)[b]	1518	40.5%
Primary caregiver for other individuals besides children (n=3286)	576	14.4%
Having 1 or more health concerns or disabilities (n=3258)	1111	36.9%
Working (n=3321)	1354	44.1%
Insurance (n=3320)		
Public	1985	55.3%
Private	975	36.2%
None/uninsured	234	5.7%
Other	126	2.9%
**Family Characteristics**		
Household size	3321	4.0 (3.0, 5.0)
**Parental Healthcare Experiences**		
First time stay at a Ronald McDonald House (n=3324)	2141	61.1%
Having more than 1 child receiving hospital care (n=3303)	223	5.9%
Ronald McDonald House family-centered services, mean (SD)[c]	3283	6.3 (1.0)

[a]Data are unweighted n (weighted %), except household size, which is weighted median (IQR), and Ronald McDonald House family-centered services, which is weighted mean (SD).

[b]Sometimes or always needs help understanding healthcare providers say or when reading health information.

[c]Ronald McDonald House family-centered services mean score ranges from 1 (least) to 7 (most).
